# Superhydrophobic engineering materials provide a rapid and simple route for highly efficient self-driven crude oil spill cleanup†

**DOI:** 10.1039/c8ra07913g

**Published:** 2018-11-14

**Authors:** Hongbo Xu, Shulong Bao, Liuting Gong, Renping Ma, Lei Pan, Yao Li, Jiupeng Zhao

**Affiliations:** MIIT Key Laboratory of Critical Materials Technology for New Energy Conversion and Storage, School of Chemistry and Chemical Engineering, Harbin Institute of Technology 150001 Harbin China jpzhao@hit.edu.cn +86 451 86403767 +86 451 86403767; Beijing Institute of Space Mechanics and Electricity 100094 Beijing China; Center for Composite Material, Harbin Institute of Technology 150001 Harbin China Yaoli@hit.edu.cn; Beijing Vocational College of Labour and Social Security 102200 Beijing China

## Abstract

Traditional superhydrophobic material use depends on two processes: creating a rough structure on a material surface and modifying the rough surface with low surface energy materials. However, common preparation methods are time-consuming, complex and cost-ineffective. Furthermore, these methods usually rely on chemicals, and evidently that will restrict mass preparation and application of superhydrophobic materials. This study reports a simple polypropylene (PP) solution-based process for producing PP hierarchical structures on commercial copper mesh (low surface energy materials), without modifying the low surface energy materials. The hierarchical structures of copper meshes, surface modified with PP, can be rationally controlled by optimizing the PP concentration. The obtained copper mesh showed contact and rolling off angles of 162° and 7°, respectively. Importantly, no significant performance loss was observed after the superhydrophobic copper meshes were continuously and drastically rinsed with 3.5 wt% NaCl solution, or repeated tearing with an adhesive tape for more than 30 cycles, indicating its good durability. After surface modification with PP particles, the copper mesh exhibits both excellent superhydrophobicity and superoleophilicity. Additionally, the as-prepared copper mesh can self-float on water surface when deformed into a “miniature boat” shape. Meanwhile, self-driven spilled oil cleanup was achieved using a superhydrophobic copper mesh-formed miniature boat. The miniature boat can realize energy conservation as well as high efficiency. The cleanup rate of the boat is as high as 97.1%, demonstrating its great potential in environmental remediation applications.

## Abstract 2

Traditional superhydrophobic material use depends on two processes: creating a rough structure on a material surface and modifying the rough surface with low surface energy materials.

## Graphical abstract

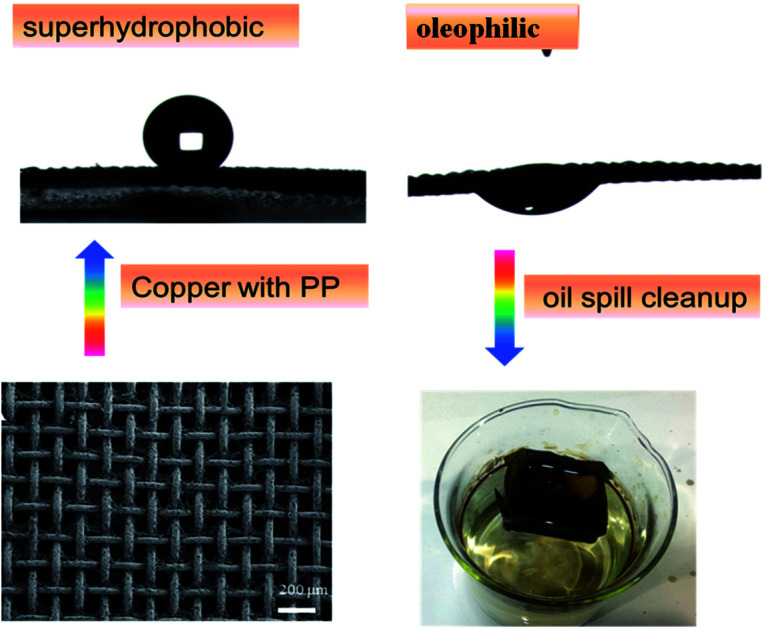

## 1. Introduction

With the increasing consumption of crude oil and its derivatives, oil spills during production and transportation happen frequently,^1,2^ which lead to a great loss of energy, severe environmental pollution and consequent ecological problems.^3–5^ Conventional methods including centrifuges, oil skimmers, depth filters, flotation and sedimentation are useful for immiscible oil/water mixtures separation, but holdbacks such as low efficiency of separation and high energy consumption severely restrict their application.^6^

Combined with superhydrophobicity and oleophilicity, materials can exhibit certain potential to separate oil/water mixtures.^7,8^ When applied to a surface, the wetting behaviour of water from sheeting and complete wetting on superhydrophilic surfaces to spherical droplet formation on superhydrophobic surfaces give inspiration to obtain solution for oil/water separation.^9–17^ Due to their efficient ability in selective absorption/separation, and favourable recyclability, superhydrophobic materials with porous structures, such as sponges, inorganic or metal fabrics and meshes, have gained broad attention for oil–water separation.^18–24^ Oil would sheet over the surface of these materials, whereas water will form droplets rendering these materials capable of separating water/oil mixtures.^25,26^ The merit of superhydrophobic meshes in the separation of water/oil mixtures is that they allow oils to go through their pores while repressing water on the surface.^27,28^

Currently, many methods have been demonstrated and a number of techniques have been exploited to produce superhydrophobic surfaces on numerous substrates including the template method,^29^ sol–gel process,^30^ self-assembly technique,^31,32^ electro-deposition,^33^ electro-spinning,^34^ hydrothermal synthesis,^35^ chemical vapour deposition,^36^ spray coating^37^ and so on. Materials fabricated using the above-mentioned methods can realize oil/water separation efficiently, however, the production processes that rely on specific equipment, are commonly time-consuming and cost-ineffective. Furthermore, they require chemicals including strong acid, strong alkali, and expensive reagents (with low surface energy) such as alkyl polysilane, alkyl sulfhydryl, and fluorosubstituted alkane. These requirements severely impede large-scale production and application. Thus, it is highly demanding to develop facile, inexpensive, and environmentally friendly strategies for producing functional absorbent materials. Nowadays, owing to low cost, low density, fascinating flexibility, high mechanical stability and other advantages, metal meshes have been considered as next generation potential material in oil/water separation.^38–40^ In particular, superhydrophobic metal meshes have gained a hot spot for the following reasons: the mesh can work as a filter and provide sufficient room for separating oil/water mixtures, meanwhile, absorbent materials on the mesh can selectively absorb the oil. However, time-consumption, complex operation and cost-ineffectiveness are main holdbacks in preparation processes such as hydrothermal treatment, oxygen plasma pre-treatment, and vapour phase deposition. Thus, pursuing a facile and time-saving method to produce functional absorbent materials for oil/water separation and spilled oil cleanup is evidently of great importance.^41–44^

In our study, a novel superhydrophobic copper mesh-based separation system was developed for the cleanup of water and crude oil spills. The fabrication process only needs several seconds with simple ingredients at room temperature, which makes it not only economical and universal but also time-saving. Specifically, copper meshes with a series of pore diameters were coated with rough PP layers. The present method shows the advantages in many aspects such as facile operation, low costs, short production times, and environmental safety. Thus, this method can be well adopted to fabricate desired PP hierarchical structures on copper mesh under mild conditions. Water contact and rolling off angles of around 162° and 7°, respectively, were realized on the as-prepared copper meshes. Moreover, after continuous rinsing with 3.5 wt% NaCl solution or repeated tearing with an adhesive tape for more than 30 cycles, the copper meshes retained good superhydrophobic performances. The miniature boat by superhydrophobic copper meshes can self-float on water surfaces and automatically recycle crude oil spills with a cleanup rate of up to 97.1%.

## 2. Experimental section

### 2.1 Materials

All solvents and chemicals were of reagent quality and were used without further purification. Hydrochloric acid (HCl), nitric acid (HNO_3_), acetone, anhydrous ethanol, xylene, sodium chloride (NaCl) and sodium hydroxide (NaOH) were purchased from Sinopharm Chemical Reagent Beijing Co., Ltd. (China). The crude oil was provided by the Chinese Petroleum Corporation. All chemicals described in the experimental section were analytical reagents. PP was purchased from Sigma-Aldrich and copper meshes were obtained from Pengda Business Company. The diameters of the copper meshes were 43 μm (350 mesh), 83 μm (180 mesh), 125 μm (120 mesh), 187 μm (80 mesh), 250 μm (60 mesh), and 500 μm (30 mesh).

### 2.2 Fabrication of a superhydrophobic and oleophilic copper mesh

**Fig. 1:**
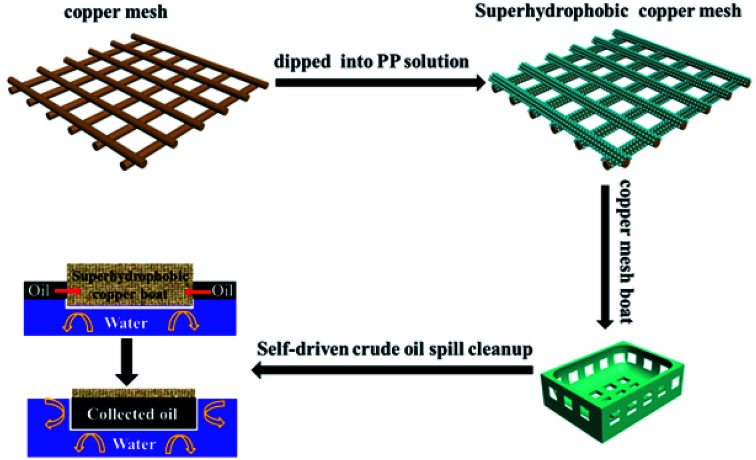
Schematic illustration of the fabrication of a superhydrophobic copper mesh with PP and a superhydrophobic boat made from the copper mesh for self-driven oil spill cleanup.

The copper mesh (size 5 cm × 5 cm) was cleaned ultrasonically in acetone, ethanol, water and 0.05 mol l^−1^ HNO_3_ solution sequentially. This procedure thoroughly removed surface impurities and enhanced the adhesion between the copper mesh and the PP films. As shown in Fig. 1, different amounts of PP (1.5 g, 2 g, 2.5 g, 3 g, 3.5 g) were added to xylene (100 ml) and stirred for 12 h at 130 °C. The cleaned copper mesh was immersed in PP solution to deposit PP hierarchical structures. The resultant copper mesh films were washed in acetone and dried in nitrogen to achieve superhydrophobicity and oleophilicity.

### 2.3 Measurement of stability of the superhydrophobic copper mesh

The chemical and mechanical stability of the superhydrophobic copper mesh was evaluated by a continuous rinsing test and a repeated tape tearing test. The manufactured superhydrophobic copper mesh was fixed on a beaker wall, and the beaker was filled with 3.5 wt% NaCl solution. Then, the magnetic stirrer was placed in the beaker, and the copper mesh was continuously rinsed at 1500 rpm for different durations. The water contact angle of the copper mesh was recorded accordingly. In addition, the copper mesh was transferred to an adhesive tape and then torn with tape. This process was repeated, and the corresponding water contact angle was recorded after each tearing cycle.

### 2.4 Fabrication of a superhydrophobic and oleophilic “copper mesh miniature boat” for self-driven oil crude collection

As shown in Fig. 1, a copper mesh film, 5.0 cm × 5.0 cm in size, was immersed in xylene. The resultant copper mesh was then modified with PP to gain superhydrophobicity and oleophilicity. The superhydrophobic copper mesh was folded into the “miniature boat” form (size 3.4 cm × 3.4 cm × 0.8 cm). The “copper mesh miniature boat” showed excellent superhydrophobic and oleophilic properties. The superhydrophobic stability of the “miniature boat” was evaluated by measuring the cleanup weight, which reflects its long-term coating ability. For the crude oil cleanup experiment, 30 g crude oil was added to a beaker with water inside to simulate a crude oil spill. Then, the miniature boat was placed in the beaker for self-driven oil spill cleanup. The cleanup rate (CR) was calculated using the following eqn (1):1
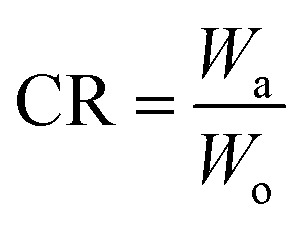
where *W*_o_, *W*_a_ are the weights of the crude oil and the oil collected by the miniature boat, respectively.

### 2.5 Characterizations of as-prepared copper meshes

The surface morphologies were characterized using scanning electron microscopy (SEM, SUPRA-55, ZEISS, Germany). The original and PP-decorated copper meshes were sputtered with gold (thickness of 5 nm). Water contact angles were measured at room temperature by a high speed digital camera (JY-PHa 2012D2, Beijing Dingsheng Digital Technology Equipment Co., Ltd., Beijing, China), and more than five repeated measurements were conducted for each sample. For the water sliding angle measurements, a 5 μl liquid droplet was used. Additional details of this measurement process can be found in the previous study.^41^

## 3. Results and discussion

Fig. 1 is the schematic illustration of the preparation of the superhydrophobic copper mesh with PP and the fabrication of a superhydrophobic boat derived from the copper mesh for self-driven oil spill collection and cleanup. First, the PP polymer was coated on the copper mesh *via* immersion and deposition in xylene solution at a high temperature (about 130 °C), where the formation of PP nanoparticles is completed within 10 min. After drying, the mesh is exposed to air at room temperature (about 25 °C). As it cools, the mesh surface gradually changes from brown, the colour of copper, to light white, which indicates that PP nanoparticles have been generated and adhered to the strings of copper mesh. Several advantages can be seen in this approach. The whole process is completed in xylene solution with PP in the absence of chemicals with low surface free energy such as alkyl polysilane, alkyl sulfhydryl, or fluorosubstituted alkane, which indicates lower costs. Furthermore, the route is facile and no complex techniques or special equipments are involved; thus, industrial mass manufacture of superhydrophobic nanostructures on copper mesh surface is highly practical. Self-driven oil spill cleanup was also achieved using a miniature boat formed by superhydrophobic copper mesh. The miniature boat could realize energy conservation as well as high efficiency.

**Fig. 2:**
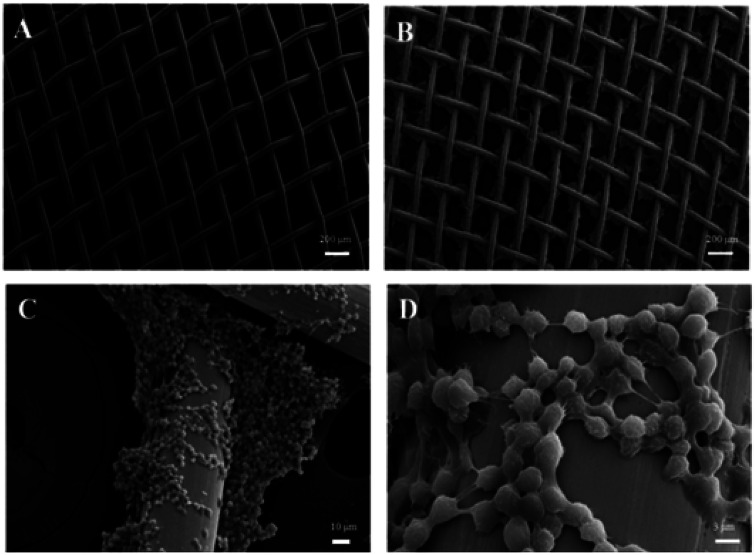
(A) SEM image of the pristine copper mesh; (B–D) SEM image and high-magnification SEM image of PP hierarchical structures (prepared with PP 25 mg ml^−1^) on copper mesh.

SEM images were used to investigate the morphology of the copper meshes before and after immersion in the PP solution (shown in Fig. 2A and B). The pristine copper mesh with no other treatment has a smooth surface (Fig. 2A). After immersion in PP solution (25 mg ml^−1^) at 30 °C for 10 min, large amount of regular and hierarchical structures are generated on the surface of the copper mesh (Fig. 2B). The corresponding high magnification SEM image illustrates that the hierarchical structure is formed by nanoparticles with an average size of about 200 nm (Fig. 2C and D), and most of the nanoparticles aggregate into microparticles of about 4 μm. With this mode of aggregation, the nanoparticles, microparticles and micrometer-sized copper wires form a hierarchical architecture with dual-scale roughness, which enhances the superhydrophobicity of the copper mesh.

All the PP-decorated copper meshes displayed rough surface microstructures at different PP concentrations similar to the 25 mg ml^−1^ PP-decorated copper meshes (Fig. S1†); however, these PP-decorated copper meshes showed different levels of wettability. Fig. S2† shows CA for copper meshes of 350, 180, 120, 80, 60, and 30 mesh counts with different PP concentrations. Evidently, all the PP-decorated copper meshes showed hydrophobicity, as the CAs were above 130°. The CAs of the PP-decorated copper meshes can be controlled by varying the PP concentration and mesh count. It can be seen that the CAs are all above 150° when the PP concentration is higher than 25 mg ml^−1^. In order to assure the uniformity of copper mesh diameters and lower the cost, the optimal concentration is 25 mg ml^−1^. Fig. S3A–F† shows the SEM images of copper mesh structures obtained with PP concentration of 25 mg ml^−1^ and with different mesh numbers, which indicates that they have similar structure with that in Fig. 1B.

**Fig. 3:**
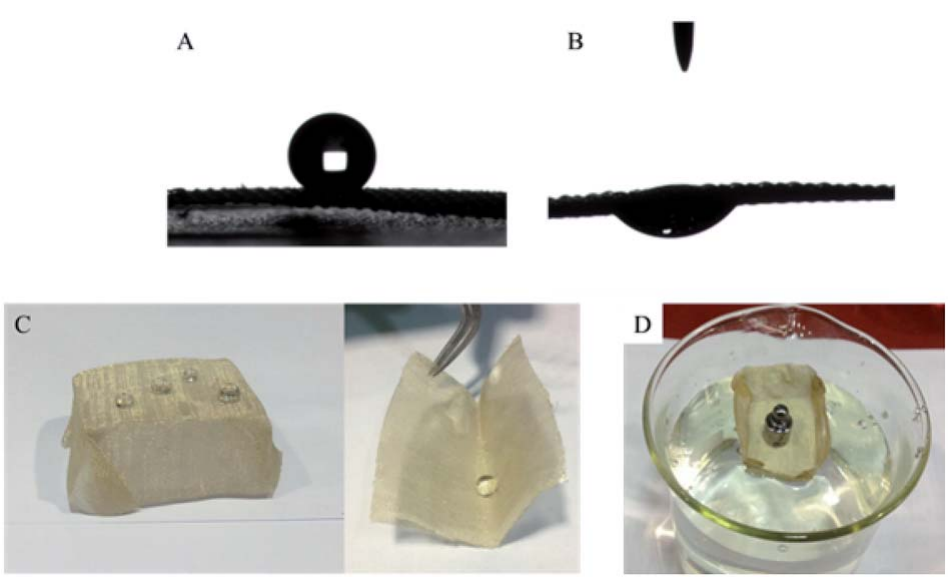
Photographs of the water droplet with a contact angle of (162.0 ± 1.4)° (A) and an oil droplet with a CA of nearly zero (B) on as-prepared superhydrophobic and superoleophilic material; (C) water CA of a superhydrophobic copper mesh at different places. (D) Oblique view of the long-term flotation of the superhydrophobic miniature boat loaded with four nuts (15 g).

**Fig. 4:**
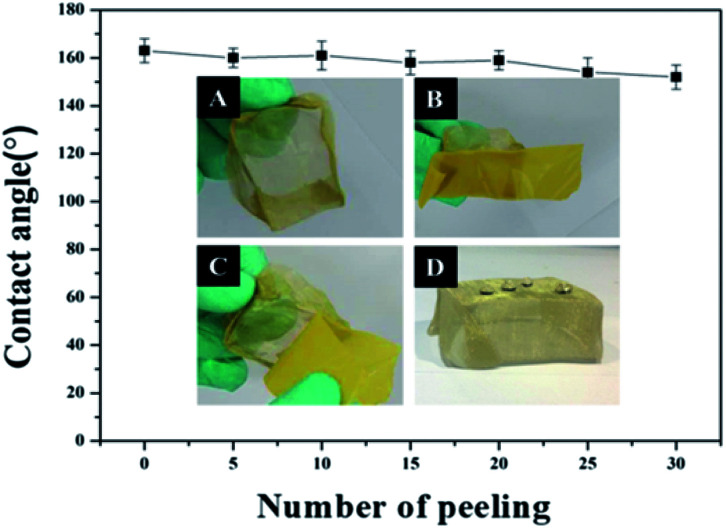
Water CA of superhydrophobic fabric repeatedly torn by adhesive tape. The inset depicts the tearing process. (A) A miniature boat; (B) the miniature boat with an adhesive tape, (C) the miniature boat with the adhesive tape peeled off; (D) water droplets on the mini boat after peeling off the adhesive tape.

**Fig. 5:**
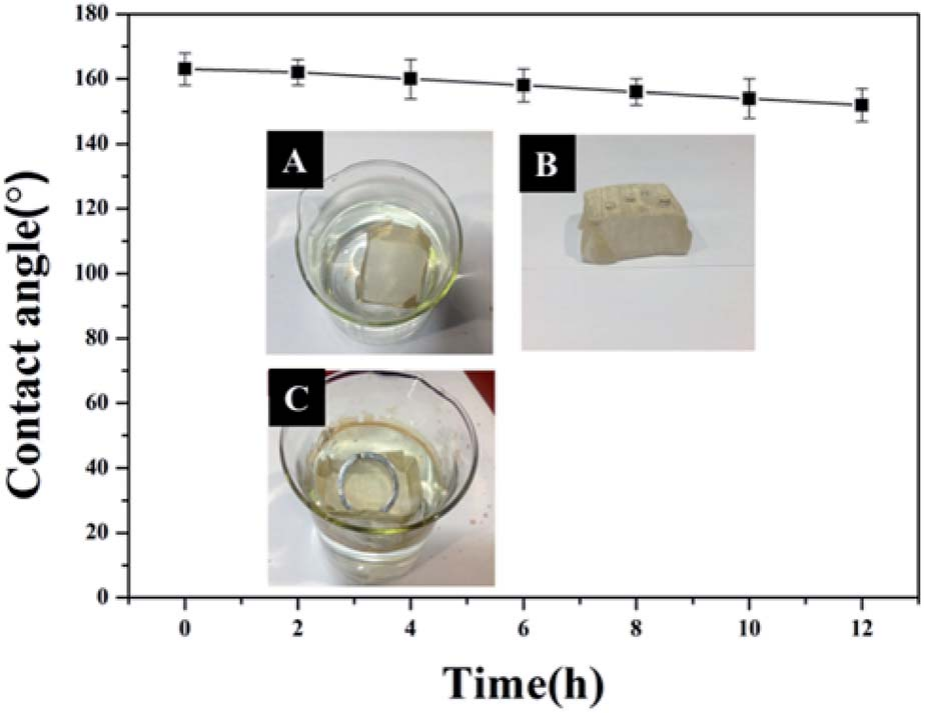
Water contact angles of a superhydrophobic miniature boat continuously rinsed with 3.5 wt% NaCl solution for different durations. The inset depicts the process of the rinse.

**Fig. 6:**
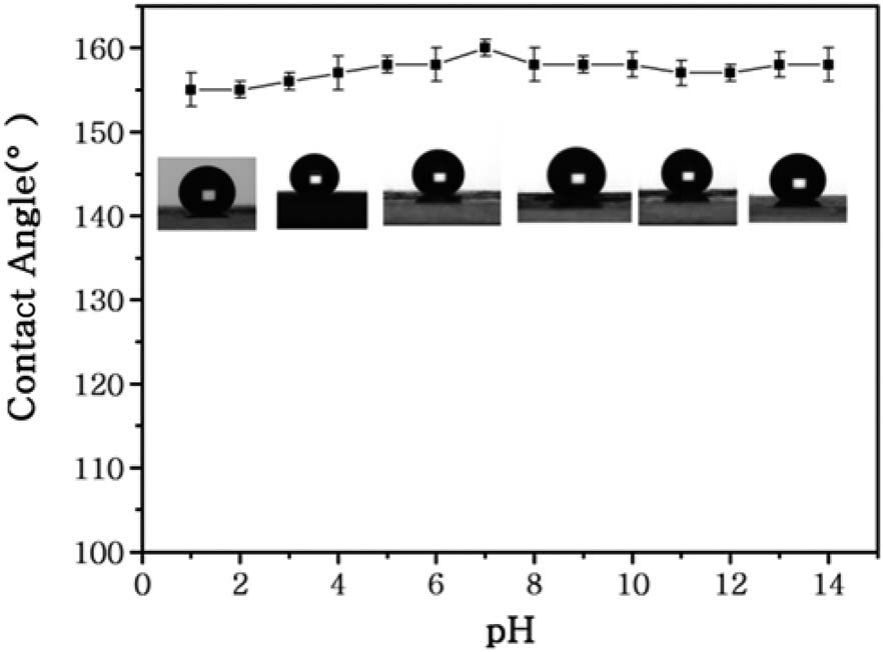
Water contact angles of a superhydrophobic miniature boat at different pH values. The insets show the corresponding photographs of the water CA.

Due to its excellent superhydrophobicity (WCA of about 160°), copper mesh-120 was chosen for further investigation. The digital photographs of water droplets and an oil droplet on the superhydrophobic copper mesh are displayed in Fig. 3A and B, respectively. Water droplets can stably remain on the surface of the boat-shaped copper mesh and maintain a spherical shape, while the oil droplets spread rapidly. The photograph (shown in Fig. 3C) clearly shows the wetting behavior of the water droplets on the superhydrophobic miniature boat. Interestingly, the superhydrophobic miniature boat can still float freely on water when loaded with one weight (20 g), indicating its outstanding superhydrophobicity (Fig. 3D). When different materials were applied on the substrate, PP hierarchical structures with different sizes and height were fabricated, which exhibits an admirable super-hydrophobic performance. For instance, the water droplets can stably remain on the surface of a stainless steel mesh with PP having a spherical shape. This displays the excellent super-hydrophobic performance of steel mesh with PP. The spherical-shaped water droplets was also obtained on other material surfaces with PP (see Fig. S4†). This demonstrated that the method can be widely applied to a variety of materials, fabricating super-hydrophobic surfaces. The stability of the superhydrophobic copper mesh was evaluated using 3.5 wt% NaCl solution to simulate ocean water, to test its water resistance under continuous rinsing and an adhesive tape to simulate external force, which will test its force resistance under repeated tearing. After rinsing for a long time of up to 8 h (Fig. 4) or a 30-cycle tear test with adhesive tape (Fig. 5), the water CA remained above 150°. The slight decline indicates that the material possesses outstanding stability. Fig. 6 displays the dynamic CA measurements on superhydrophobic copper mesh modified with PP at pH values from 1 to 14; insets are corresponding photographs of the water CA. This superhydrophobic copper mesh, which has excellent stability, could provide promising materials for tackling environmental and energy issues such as crude oil spill cleanup.

**Fig. 7:**
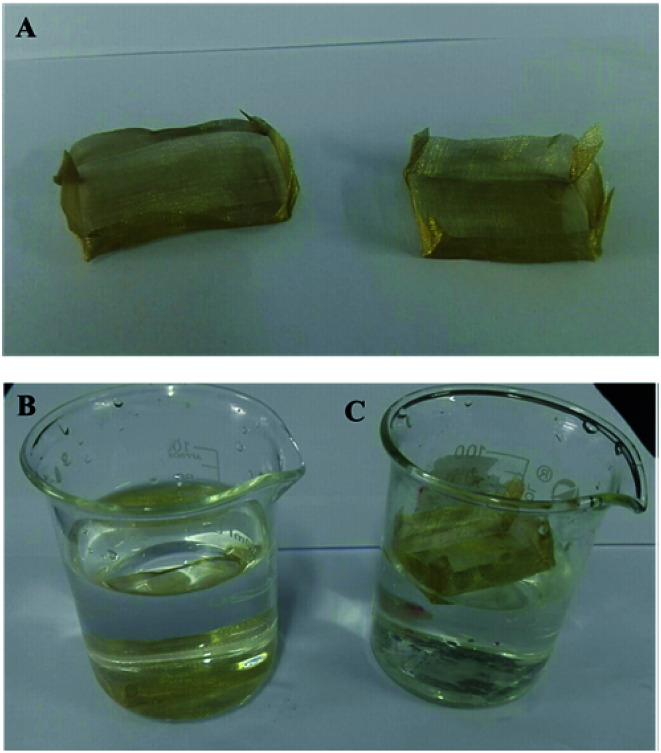
(A) Photograph of a miniature boat fabricated from copper mesh and superhydrophobic copper mesh; (B) a miniature boat fabricated from pristine copper mesh sinks in water; (C) a miniature boat fabricated from superhydrophobic copper mesh floats in water.

Although tremendous efforts have been put into preparation of superhydrophobic materials and fabrication of devices for oil spill cleanup, extra power input is still necessary to achieve the continuous separation of oil and water. In order to achieve an automatic collection of oil without any power input, we propose the use of a miniature superhydrophobic boat, as superhydrophobic porous materials can float freely on water. As shown in Fig. 7A, a miniature boat is fabricated from copper mesh and superhydrophobic copper mesh. The miniature boat fabricated from pristine copper mesh cannot float on water (Fig. 7B), while the miniature boat made from superhydrophobic copper mesh can float freely on water (Fig. 7C).

**Fig. 8:**
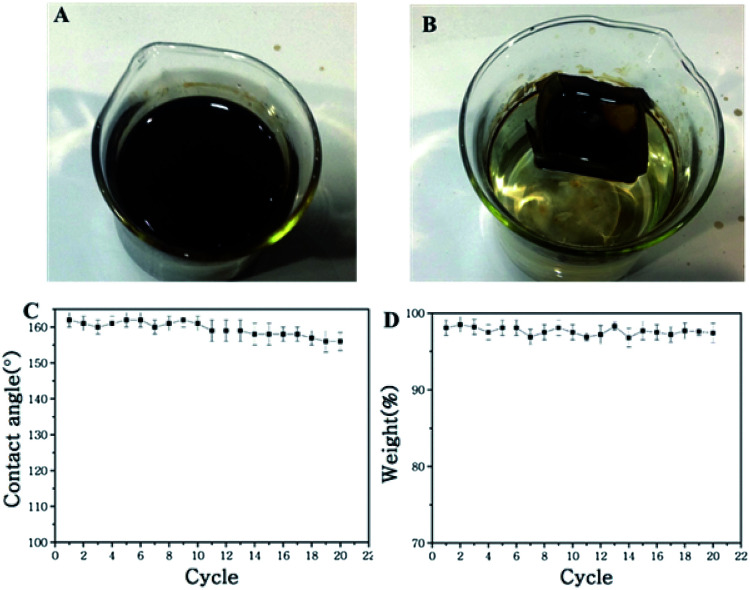
(A and B) The process of self-driven crude oil spill cleanup of diesel oil from water using the as-prepared miniature boat; (C) water CAs of a reused miniature boat; (D) the crude oil cleanup rate of the reused miniature boat.

Fig. 8 illustrates the process of a self-driven crude oil spill cleanup simulation. The water is covered with crude oil spill (30 g), and the miniature boat was placed the surface of water. Under permeation, the crude oil spill was absorbed and entered the miniature boat. Meanwhile, there was no observation of water permeation into the boat (Fig. 8B). In less than 1 minute, the oil absorption process was complete, and about 29.1 g of oil was collected. The crude oil clean-up rate reached as high as 97.1%. Importantly, the mini boat from the superhydrophobic copper mesh can be reused for self-driven oil spill cleanup. The reused miniature boat exhibited a water CA of about 152°, which is slightly less than the original 162°, and the crude oil cleanup rate reached as high as 95% after 30 times of usage (shown in Fig. 8C and D). Meanwhile, the digital photograph (shown in Fig. S5†) clearly displays the water droplets maintaining a spherical shape on the surface of the reused miniature boat, which indicates that the copper mesh still possesses superhydrophobicity. Compared to pristine copper mesh, the CA and weight-bearing capacity of the reused copper mesh has slightly decreased, but the mesh is still capable of floating and separating oil and water (shown in Fig. S5†). This demonstrates that the present approach exhibits excellent stability and holds highly promising application in oil/water separation and offshore crude oil spill cleanup.

## 4. Conclusions

In summary, we report a facile approach to fabricate robust superhydrophobic copper meshes through one-step PP solution-immersion process. This copper mesh exhibited excellent oil/water separation properties. The morphology of the PP coatings on copper meshes was mainly affected by the concentration of PP/xylene solutions. Water contact and rolling off angles of 162° and 7°, respectively, were realized on the as-prepared copper meshes. Moreover, the superhydrophobic copper meshes can withstand continuous and drastic rinsing with 3.5 wt% NaCl solution and repeated tearing with an adhesive tape for more than 30 cycles. The results indicate that the superhydrophobic copper meshes have good mechanical stability and excellent resistance to water and external force. The as-prepared copper meshes exhibit superwetting properties, excellent mechanical durability and self-driven crude oil spill cleanup capabilities. Furthermore, the superhydrophobic boat by copper meshes can automatically recycle crude oil spills while floating freely on water with a cleanup rate of up to 97.1%, indicating great potential in environmental remediation applications. Considering the simple fabrication process, high commercial availability of copper mesh, excellent reusability, as well as good mechanical stability, this method holds highly promising application in oil/water separation and off shore crude oil spill cleanup.

## Conflicts of interest

There are no conflicts to declare.

## Supplementary Material

RA-008-C8RA07913G-s001

RA-008-C8RA07913G-s002

RA-008-C8RA07913G-s003
