# Supplementary material for: Superhydrophobic engineering materials provide a rapid and simple route for highly efficient self-driven crude oil spill cleanup
Source: RSC Adv. 2018 Nov 14;8(67):38363–9. doi: 10.1039/c8ra07913g (PMC9089833; doi:10.1039/c8ra07913g)
Supplement: RA-008-C8RA07913G-s001 [file RA-008-C8RA07913G-s001.pdf]

Electronic Supplementary Material (ESI) for RSC Advances.
This journal is © The Royal Society of Chemistry 2018

Supporting Information

Superhydrophobic Engineering Materials via a Rapid and Simple Route for Highly Efficient Self-driven Crude Oil Spill Cleanup

Hongbo Xu[1], Shulong Bao[2], Liuting Gong[1], Renping Ma[4], Lei Pan[1], Yao Li*[3], Jiupeng Zhao*[1]

1. Address: MIIT Key Laboratory of Critical Materials Technology for New Energy Conversion and Storage, School of Chemistry and Chemical Engineering, Harbin Institute of Technology, 150001, Harbin, China. Fax: +86 451 86403767; Tel: +86 451 86403767.

2. Address: Beijing Institute of Space Mechanics and Electricity, 100094, Beijing, China

3. Address: Center for Composite Material, Harbin Institute of Technology, 150001, Harbin, China

4. Address: Beijing Vocational College of Labour and Social Security, 102200, Beijing, China.

Emails: Yaoli@hit.edu.cn or jpzhao@hit.edu.cn

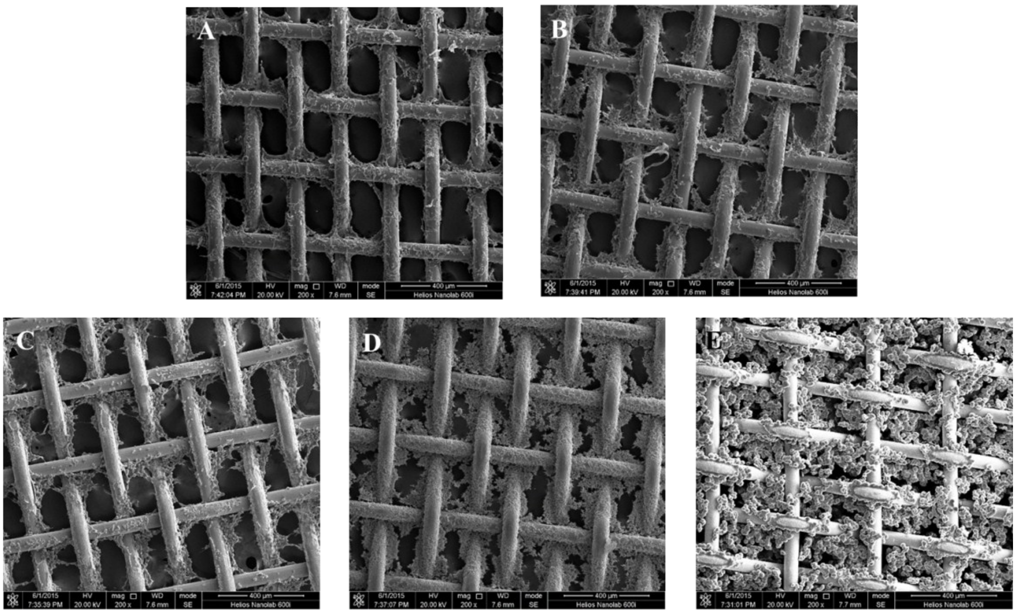

Figure S1. SEM images of PP decorated copper mesh with (A) 15 mg/ml, (B) 20 mg/ml, (C) 25mg/ml, (D) 30 mg/ml, (E) 35 mg/ml of PP, respectively.

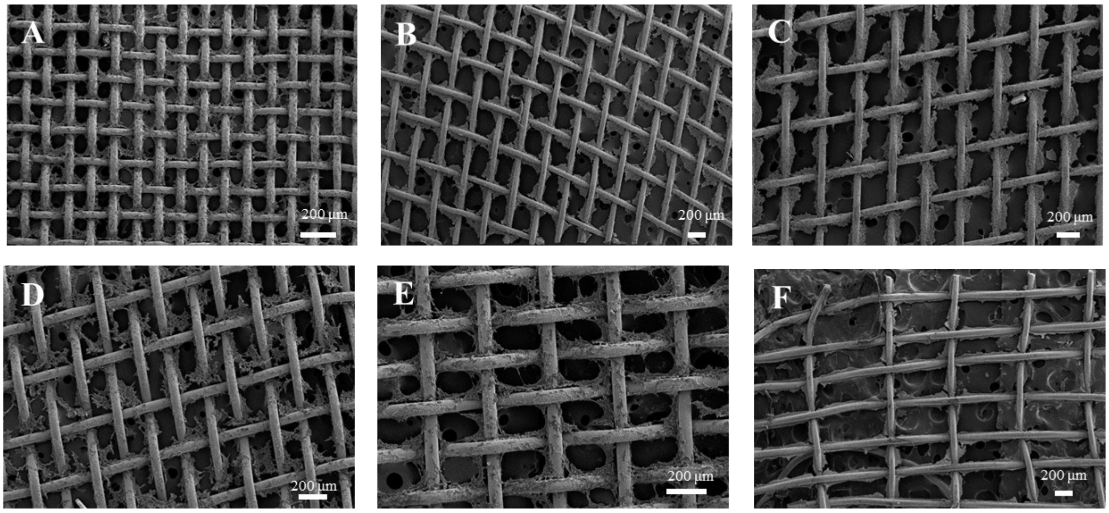

Figure S2. SEM images of PP decorated copper mesh with (A)350-copper mesh, (B)180-copper mesh, (C)120-copper mesh, (D)80-copper mesh, (E)60-copper mesh, (F)30-copper mesh at 25 mg/ml of PP.

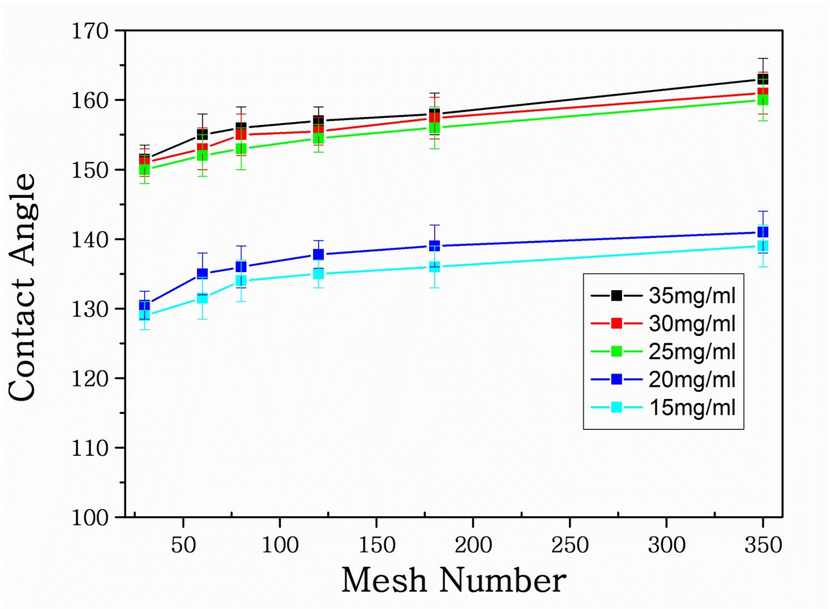

Figure S3. Water contact angles of PP decorated copper mesh.

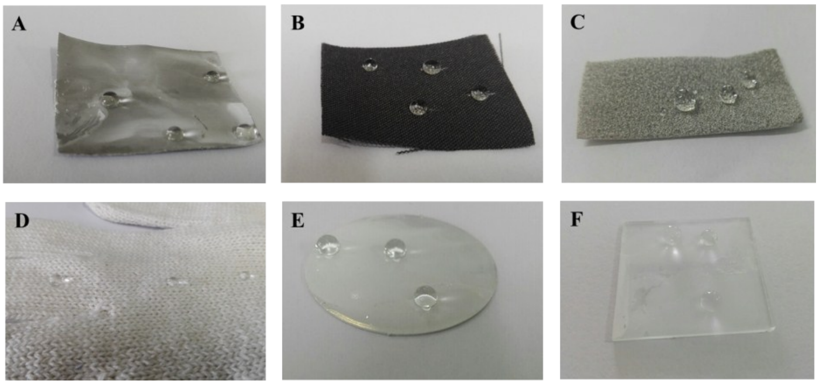

Figure S4. The photographs of the water droplets on different material (A) stainless steel mesh, (B) carbon fiber cloth, (C) nickel foam, (D) nylon gloves, (E) aluminum foil, (F) glass slide.

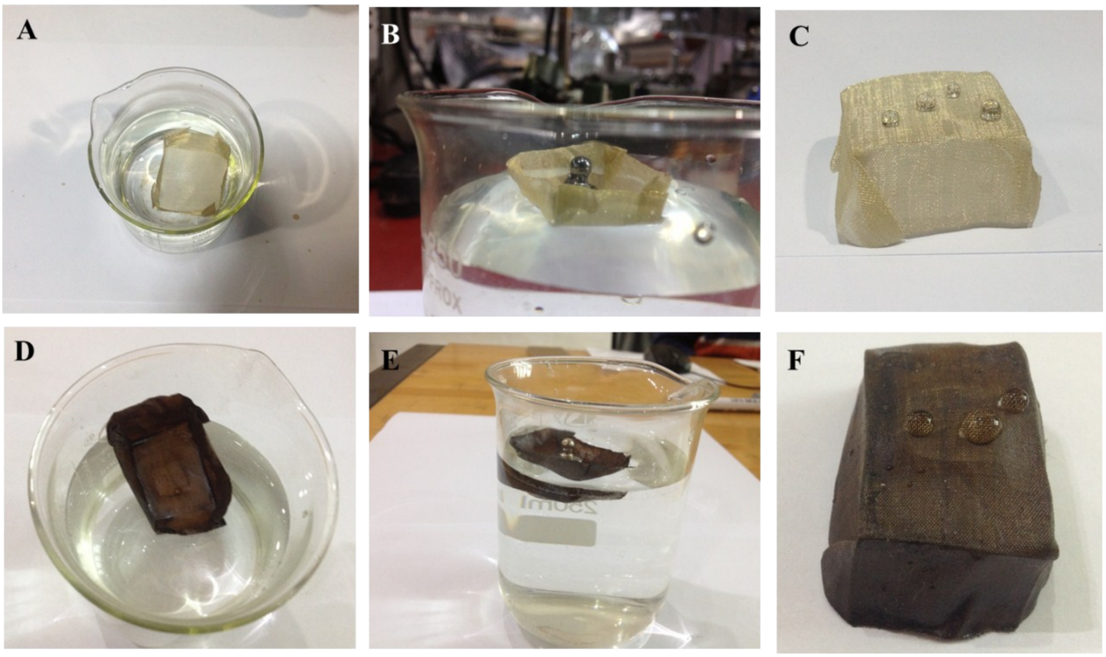

Figure S5. The photographs of the water droplets on unused miniature boat (A, B, C) and reused miniature boat (D, E, F).

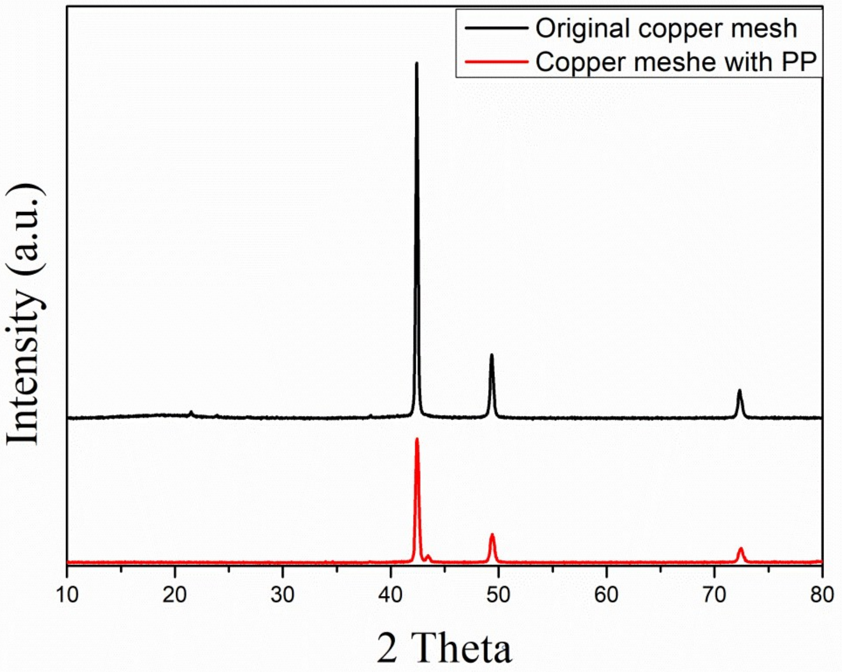

Original copper mesh
Copper meshe with PP
Intensity (a.u.)
10
20
30
40
50
60
70
80
2 Theta

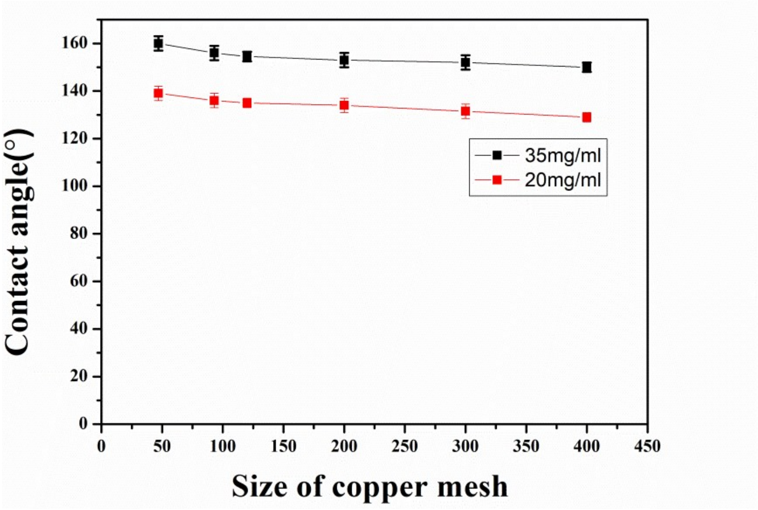

Figure S7 Water contact angles of different porous size copper mesh with PP

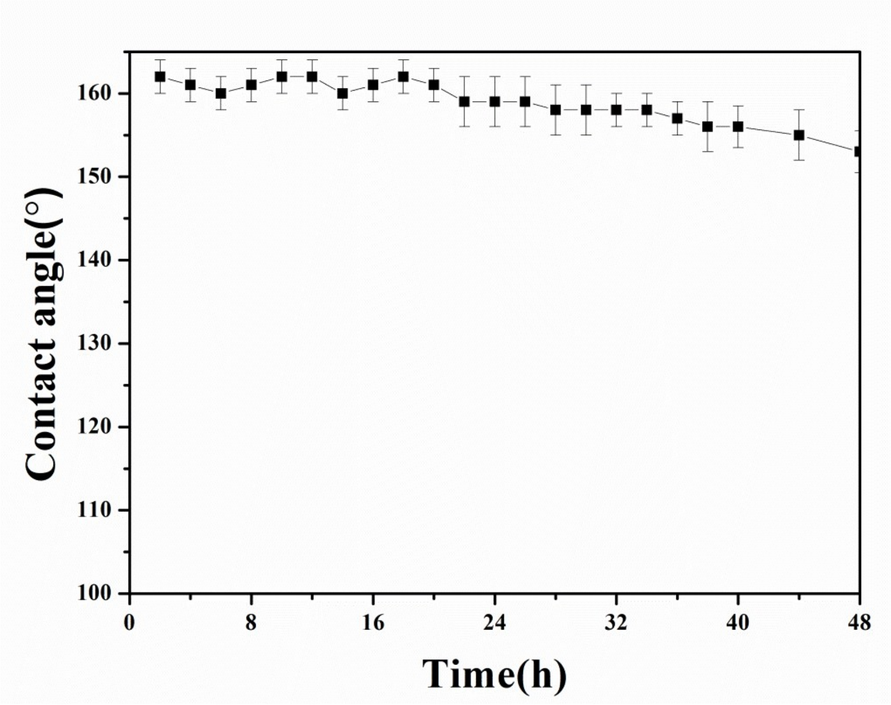

Figure S8. Water contact angles of superhydrophobic coper mesh by 1M HCl solution for different durations.
